# The Measles Vaccination Narrative in Twitter: A Quantitative Analysis

**DOI:** 10.2196/publichealth.5059

**Published:** 2016-01-04

**Authors:** Jacek Radzikowski, Anthony Stefanidis, Kathryn H Jacobsen, Arie Croitoru, Andrew Crooks, Paul L Delamater

**Affiliations:** ^1^ Center for Geospatial Intelligence George Mason University Fairfax, VA United States; ^2^ Center for Geospatial Intelligence Department of Geography and Geoinformation Science George Mason University Fairfax, VA United States; ^3^ Department of Global and Community Health George Mason University Fairfax, VA United States; ^4^ Department of Computational and Data Sciences George Mason University Fairfax, VA United States; ^5^ Department of Geography and Geoinformation Science George Mason University Fairfax, VA United States

**Keywords:** social media, health narrative, geographic characteristics, data analysis, health informatics, GIS (geographic information systems)

## Abstract

**Background:**

The emergence of social media is providing an alternative avenue for information exchange and opinion formation on health-related issues. Collective discourse in such media leads to the formation of a complex narrative, conveying public views and perceptions.

**Objective:**

This paper presents a study of Twitter narrative regarding vaccination in the aftermath of the 2015 measles outbreak, both in terms of its cyber and physical characteristics. We aimed to contribute to the analysis of the data, as well as presenting a quantitative interdisciplinary approach to analyze such open-source data in the context of health narratives.

**Methods:**

We collected 669,136 tweets referring to vaccination from February 1 to March 9, 2015. These tweets were analyzed to identify key terms, connections among such terms, retweet patterns, the structure of the narrative, and connections to the geographical space.

**Results:**

The data analysis captures the anatomy of the themes and relations that make up the discussion about vaccination in Twitter. The results highlight the higher impact of stories contributed by news organizations compared to direct tweets by health organizations in communicating health-related information. They also capture the structure of the antivaccination narrative and its terms of reference. Analysis also revealed the relationship between community engagement in Twitter and state policies regarding child vaccination. Residents of Vermont and Oregon, the two states with the highest rates of non-medical exemption from school-entry vaccines nationwide, are leading the social media discussion in terms of participation.

**Conclusions:**

The interdisciplinary study of health-related debates in social media across the cyber-physical debate nexus leads to a greater understanding of public concerns, views, and responses to health-related issues. Further coalescing such capabilities shows promise towards advancing health communication, thus supporting the design of more effective strategies that take into account the complex and evolving public views of health issues.

## Introduction

The Internet has provided health informatics with a new lens to study health-related issues. For example, Internet-based biosurveillance and digital disease detection approaches have been used to gain insight into emerging disease threats [1,2]. A main focus of earlier efforts was placed on identifying the likelihood of new outbreaks based on observations of increased mentions of disease-related terms. For example, Google Flu Trends maps the number of search engine queries about the word influenza and related terms and predicts emerging outbreaks as changes in the frequency of such queries [3]. While those types of approaches have been successfully applied to tracking and monitoring disease outbreaks, the emergence of social media enables researchers to move beyond this by incorporating valuable insights about people’s opinions and perspectives on health issues.

In this paper, we present a case study that showcases the emergence of a health narrative from social media content, focusing on the reaction on Twitter to the recent outbreak of measles. In the context of this study, we use the term “health narrative” to refer to the structure of the discussion as it is observed on Twitter. This structure is characterized by associations. Associations between words reveal the semantic composition of the discussion, exposing themes and clusters of topics, and even term connotations. Associations between cyber contributions and their corresponding geographical space help reveal the connection between observations in the Twittersphere and current health issues that affect the general public. It has to be noted here that the structure of this narrative is implicit and emerges from the individual contributions, rather than being explicit and imposed by a certain authority.

The research objective of this paper is to explore how such health narrative structure may be discerned from individual contributions and its value. In order to pursue this goal, we use the 2015 measles outbreak as a case study and demonstrate how narrative elements are extracted from it and how they relate to the ongoing public debate regarding this issue. We show the relative impact on this process of different sources of information (namely media and authoritative health organizations) and highlight the cyber and spatial footprints of an ongoing debate regarding vaccination.

Social media provide the general public with newfound mechanisms to receive and contribute information, often in real-time. While these communities started off as cyber curiosities, participation has now reached massive levels. As of spring 2015, Twitter has nearly 300 million active users globally, and Facebook has a remarkable 1.4 billion active users [4]. According to a survey conducted by the Pew Research center in late 2014, 58% of all American adults use Facebook, 21% use Instagram, and 19% use Twitter [5]. Accordingly, these social media platforms are no longer limited to supporting the simple exchange of messages among friends. They have evolved to play a formative role in shaping global public opinion on a broad array of topics, ranging from politics [6] and entertainment [7] to science [8] and business [9].

Researchers from the health community realized early the potential offered by social media to change health-related communication patterns across the United States and the rest of the globe [10]. By their nature, social media represent a transition from one-to-one health communications between clinicians and their patients to many-to-many communications between health care providers, patients, and broader communities. They also broaden the scope of health discussions, no longer focusing exclusively on reporting disease outbreaks but also addressing health care service, with patients sharing their experiences with various health providers [11].

This transition toward interactive communication presents opportunities and challenges [12] that exceed those introduced by the traditional role of the Internet merely as a publicly accessible repository of information [13-16]. Collective discourse in social media leads to the formation of a complex narrative, conveying public views and perceptions.

With major health organizations embracing social media as a new avenue to communicate (and also harvest) health-related information to (from) the general public, advancing our understanding of the patterns of health narrative in social media is becoming essential. Terry [17] discussed how the Centers for Disease Control and Prevention (CDC) utilized Twitter in the context of the 2009 H1N1 influenza outbreak. On the same issue, Chew and Eysenbach [18] studied the use of Twitter traffic related to H1N1 for real-time content analysis and knowledge extraction in the context of infodemiology. More recent studies suggest that such analysis can even be applied not only to monitor broad epidemics [19], but also to harvest more personal content, such as reports of adverse reactions to medication [20,21].

Reflecting the strong potential of social media for health communication, in 2014 the World Health Organization (WHO) used Twitter to communicate information regarding the Ebola outbreak in West Africa. However, public opinion is formed not only as a top-down process (ie, authoritative sources such as WHO communicating their views to the general public) but also as a bottom-up process (whereby individual users establish circles of influence) [22,23]. These patterns of health narrative are complex and need to be studied in order to be better understood.

This paper contributes to this goal by presenting a study of the narrative in Twitter regarding measles vaccination in early 2015, focusing specifically on the intersection between this narrative and a grass-roots antivaccination movement. The contributions of this work are the analysis of the data for this particular case study, as well as the presentation of a broader approach to analyze such open-source data. As such, this line of inquiry has the potential to further advance health communications by improving our understanding of the mechanisms through which information is disseminated in social media.

## Methods

### Design

The objective of this analysis was to study the Twitter narrative about vaccination in the aftermath of the 2015 measles outbreak, both in terms of its cyber and physical characteristics. Toward this goal, the Twitter application program interface (API) was accessed in order to collect tweets between February 1 and March 9, 2015, using the keyword “vaccination” or its derivatives that are often encountered in social media (ie, “vaccine,” “vaccines,” “vax,” “vaxine,” and “vaxx”). These 6 variants of the term vaccination were selected following a brief study of Twitter traffic related to vaccination for a 48-hour period directly preceding our formal study. In that pre-study, these five variants were the predominant alternate versions of the word vaccination, and as such were used together with it for our subsequent formal study.

### Data Collection

The GeoSocial Gauge system prototype was used to collect data from Twitter using a user-specified set of parameters such as keywords, locations, and time [24]. This system allows researchers to retrieve the actual tweet content as well as its metadata, including information such as user name, timestamp, and location. The system also performs basic quantitative analysis of extracted data. A geosocial analytic approach was used to explore the geographical distribution of tweets as well as social network properties.

### Data Characteristics

Using these keywords, a total of 669,136 tweets were collected from across the globe. Among these tweets, 356,248 tweets (53.24% of the total) had some type of geolocation associated with them, to indicate the location of the user that posted them. A total of 6266 tweets had geolocation in the form of precise coordinates, which tends to be as accurate as few meters and is typically associated with tweets posted from users through their mobile phones. An additional 351,973 were geolocated at the level of a toponym reference (ie, at the level of a city or neighborhood). These patterns of geolocation are consistent with figures reported from other analyses. More specifically, the precisely geolocated tweets represented 0.94% (toponym reference: 52.60%) of the total number of tweets, and broader studies have reported such precisely geolocated tweets to amount to between 0.5% and 3% of the overall traffic with toponym references typically ranging from 40-70% [25].

**Figure 1:**
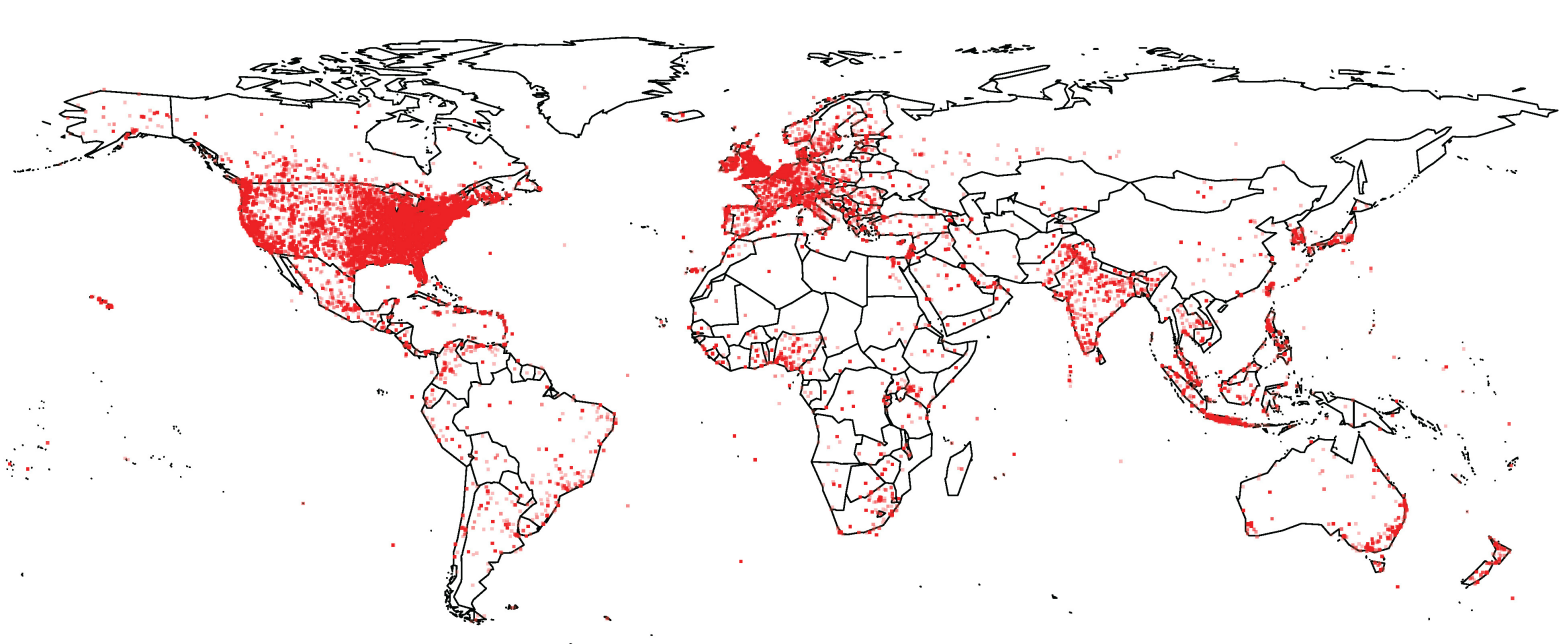
Global distribution of tweets in our data corpus.

Figure 1 shows the global distribution of the geolocated tweets in our data corpus, with 60.18% of them (214,396/356,248) originating from within the United States. Similarly, over half (54.69%, 3432/6266) of the precisely geolocated tweets originated from the United States. Table 1 summarizes the 10 countries contributing the most tweets during that period. Tweets originating from the United States dominate the data, with a volume of contribution that is one order of magnitude larger than that of the second country (Canada), and two orders of magnitude larger than the rates of the countries that round off that list. This pattern of distribution of contributions is not uncommon for Twitter, especially when it is affected by high profile events (as was the 2015 measles outbreak for our study), which tend to amplify Twitter traffic [26].

**Table 1:** The 10 countries contributing the highest number of tweets in our data corpus.

Country	Tweets (% of geolocated total), n (%)
United States	214,396 (60.18)
Canada	20,039 (5.63)
United Kingdom	15,018 (4.22)
India	9249 (2.60)
Australia	8207 (2.30)
Indonesia	2864 (0.80)
France	2492 (0.70)
Pakistan	2448 (0.69)
Germany	2370 (0.67)
Nigeria	2263 (0.63)

Regarding frequency of contributions, our data reflect a global average of just over 18,000 tweets daily, or more than 750 tweets hourly (5794 geolocated tweets originating daily from the United States); 272,795 distinct users contributed the tweet corpus. While this would indicate an average of 2.45 tweets on the subject per user, participation in social media deviates from a normal distribution and instead tends to follow power law patterns [27]: a large number of users tweet infrequently, while a small number of them are very prolific. This behavior is consistent with observed blogosphere characteristics [28] and is comparable to behavioral patterns observed in online forums [29]. In the data corpus, the median number of vaccine-related tweets per user was 5, while the three most active users contributed more than 1000 tweets each. Six of the 10 most prolific authors are notable antivaccination advocates (account handles are not reported here for privacy considerations).

### Analysis Objectives

**Figure 2:**
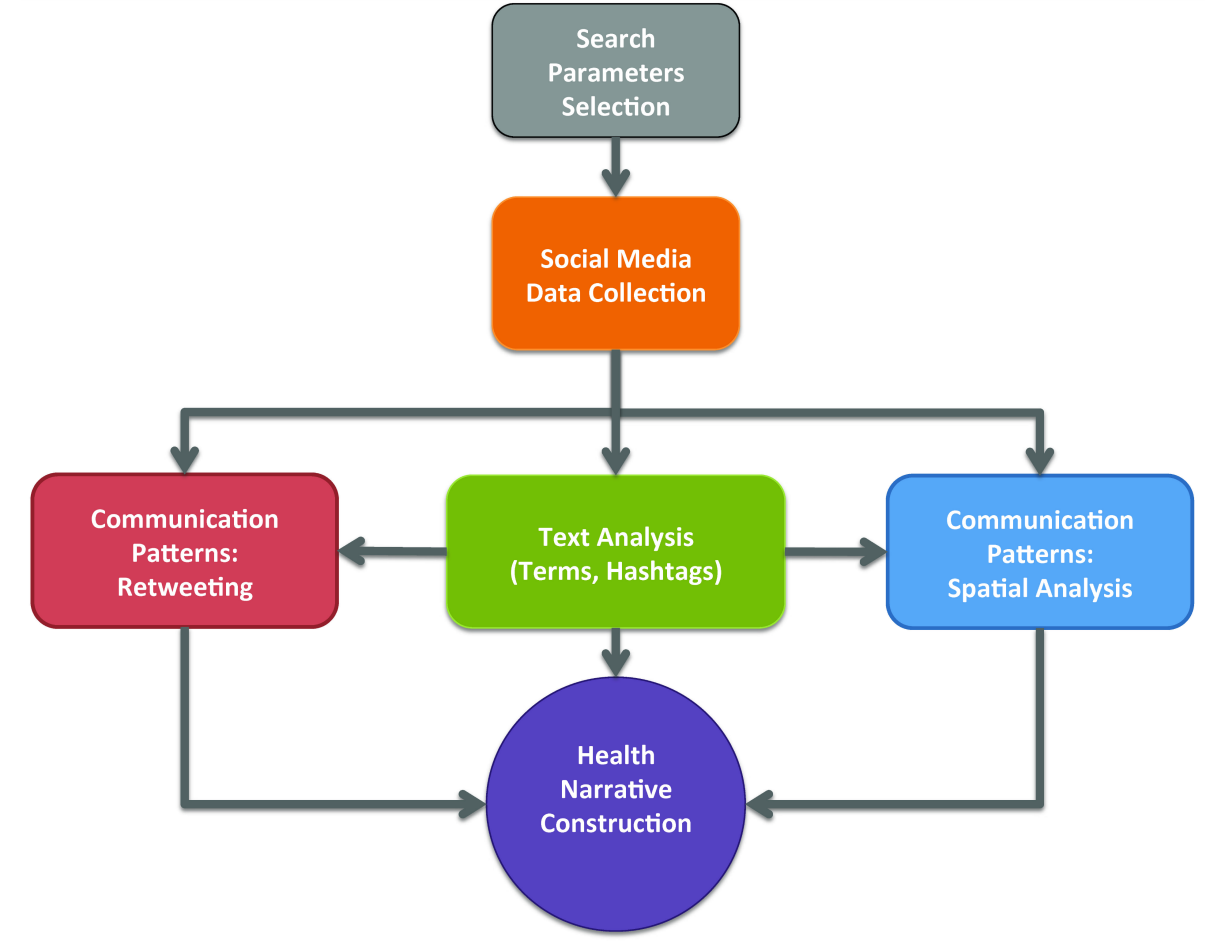
A summary of our approach.

Our primary objective was to assess the characteristics of the vaccination narrative in cyber and physical spaces. Toward this goal, our study assesses the characteristics of discussion terms that comprise the narrative in Twitter and of the communities that were involved in this discussion. Figure 2 summarizes our approach. We start with a selection of search parameters, which are typically a set of keywords and potential geographical areas of interest. Using these parameters, we access the Twitter API for data collection, harvesting tweets that include these keywords and originate from the area of interest. These tweets are then analyzed to extract terms and patterns that reveal the narrative structure. This structure comprises three dimensions: text, retweeting patterns, and spatial patterns.

Regarding text analysis, we identify dominant terms and popular hashtags, as well as their associations in the form of co-occurrences. Terms and hashtags serve as the equivalent of keywords for the overall narrative: they reflect the topics that are considered relevant and important by the general public. Their associations reveal the thematic components of the narrative structure, in the form of subthemes and contextual connotations, as they emerge from the crowd. Regarding communication patterns as they are revealed through retweeting, our primary objective is to assess the impact of various sources of information, contrasting diverse types of authoritative content (eg, health organizations and official news organizations) and grass-roots campaign arguments (with the antivaccination community views serving as a prototypical example). We are also interested in assessing the spatial patterns of communications by studying the locations from which these contributions are being made to social media. This allows us to gain insights on the debate in cyberspace as well as the connection between cyber and physical communities, and consequently between the ongoing community debates across the continental United States regarding vaccination.

## Results

### Dominant Terms

**Figure 3:**
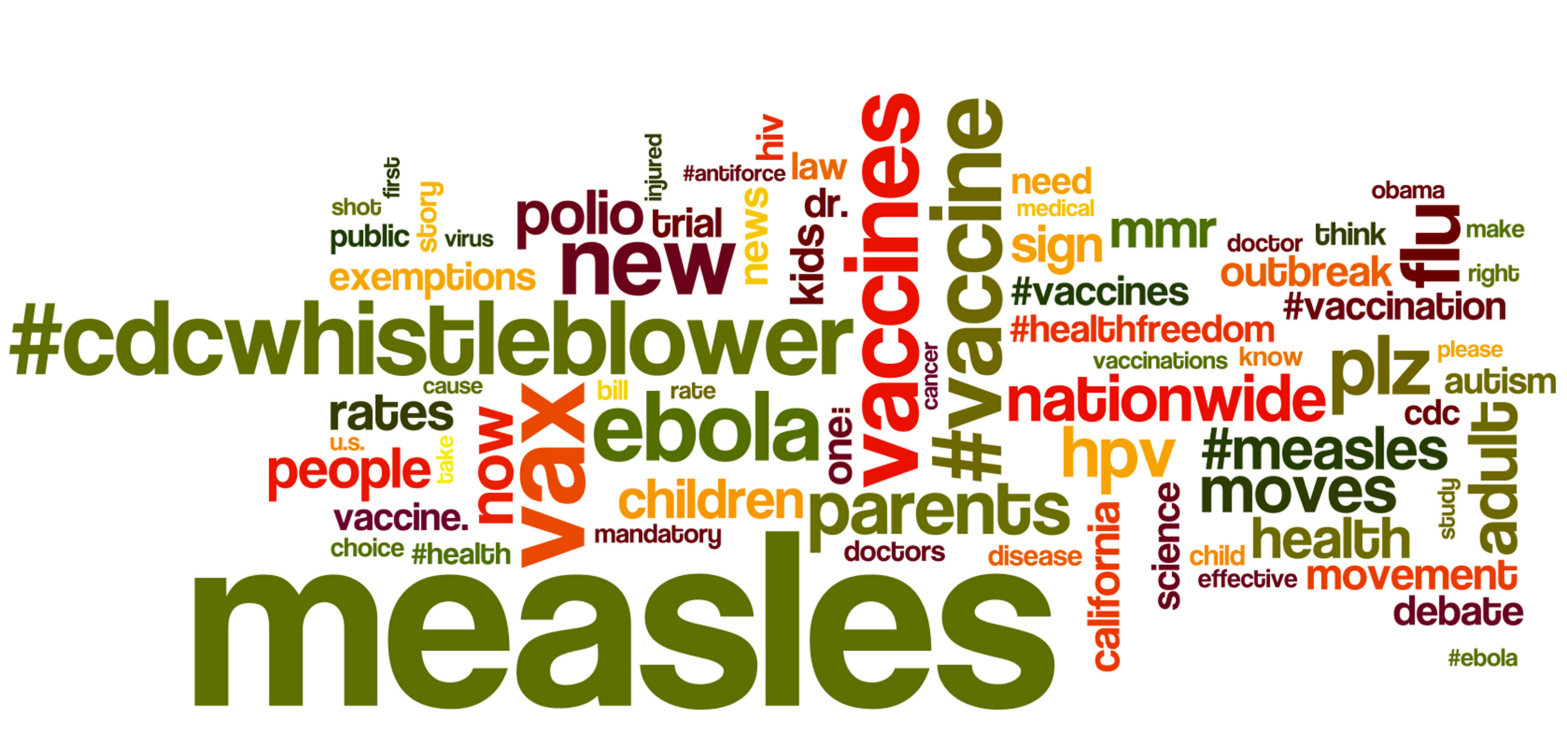
Word cloud of the 75 terms most frequently encountered in Twitter in the context of the vaccination study.

Given the design of the data collection process, all of the tweets in the data corpus for this analysis included the word vaccination or one of its derivatives. Figure 3 shows a word cloud visualization of the 75 most frequently encountered terms in the data corpus, in order to provide a general overview of the dominant narrative terms. The word cloud excludes the search words vaccine and vaccination because their very high frequency (appearing in 279,684 and 123,342 tweets respectively) would make all other data dwarf. The word cloud also excludes stop words (ie, articles, prepositions, and common verbs), as such words are common to all discussions and therefore lack semantic significance. In the word cloud, the relative size of each word is proportional to its frequency, where words in larger font are the ones more often encountered in the data corpus. In the word cloud, hashtags are treated as distinct words. For example, measles and #measles are considered as two separate terms. A hashtag reference indicates a stronger emphasis on the word, rather than the simple reference to it within the tweet text [30], so these terms have distinct uses within the Twitter discussion.

Table 2 lists the 10 most frequently encountered health-related terms in the data corpus. The list excludes vaccination and its various derivative forms, stop words as defined above, and common words such as new, now, people and against. The overall number of mentions is listed, along with the percentage of tweets in which each term was present.

**Table 2:** Ten most frequently encountered health-related terms in the data corpus.

Term	Mentions (frequency)
measles (and #measles)	82,179 (12.28%)
#cdcwhistleblower	27,876 (4.17%)
Ebola	26,273 (3.93%)
flu	22,429 (3.35%)
HPV	19,253 (2.92%)
polio	16,749 (2.50%)
health	15,546 (2.32%)
MMR	14,777 (2.21%)
#healthfreedom	10,356 (1.55%)
autism	10,101 (1.51%)

Measles was the most common term encountered in these tweets about vaccination, which is expected given that these data were collected during the US measles outbreak in early 2015. Furthermore, Ebola and HPV (human papilloma virus) are also encountered among the top terms associated with the discussion, reflecting the general interest in the media regarding vaccinations for them during that period.

It is interesting to observe that the second most popular term was #cdcwhistleblower, which emerged in August 2014 as a quick identifier to the antivaccination community of messages aligned with antivaccination views. This term did not originate from a formal organization, but instead it is one that has emerged from an online advocacy community as a means to consolidate its views and promote its perspectives. In contrast, references to official health organizations were uncommon. For example, CDC had only 9611 mentions in the data corpus, making it the 47^th^ most popular term, while WHO and NIH (National Institutes of Health) had only 351 and 330 mentions respectively and were not within the top 2000 terms in the data corpus. Accordingly, the data indicated that a bottom-up campaign (represented by #cdcwhistleblower) far outweighed the presence of official sources such as the top-down efforts of CDC and WHO. This pattern is indicative of the complex notion of authority in the information dissemination landscape of social media.

### Communication Patterns: Retweeting

Among the 669,136 tweets, 296,223 were retweets (and conversely, 372,913 were original tweets). These retweets account for 44.27% of the overall data corpus (42.20% within the United States and 45.25% overseas). This is substantially higher than reported figures regarding retweet activity in Twitter overall, whereby retweets typically account for only 30% of overall Twitter traffic [31]. Such increased retweeting patterns are comparable to ones observed in studies of Twitter traffic during elections [32], which showed that highly opinionated users tend to retweet more than their less opinionated counterparts. Vaccination appears to be a “political” or partisan topic among Twitter uses, and high levels of retweeting activity may reflect high levels of activism among the participants.

Retweeting is part of the process of community formation and information dissemination in Twitter [33]. Similar to the patterns of participation, the pattern of retweeting has been shown to be highly skewed [34], with the large majority of tweets receiving one to two retweets each and very few receiving high numbers. The data corpus was consistent with this pattern, with a median number of 1 retweet per tweet, and a maximum value of 3399 retweets of a single tweet (see Textbox 1).

**The five most retweeted messages.**

1: “The Disneyland Measles Outbreak Is A Turning Point In The Vaccine Wars http://t.co/qHVBxyvDMF via @username1” (3399 retweets in the data corpus)

2: “@username2 @username3 Parents can delay timing of vaxx if they want more time between shots. Should be done by time they enter school.” (2899 retweets)

3. RT @username4: Anti-vax dad is cool with his kid fatally infecting others, also blames leukemia on vaccines. http://t.co/XuSkaK9SdQ http:/...” (2002 retweets)

4. RT @username5: Vaccination isn’t a private choice but a civic obligation.”  F****’ A right. http://t.co/pNj5w7fp9t” (1630 retweets)

5. RT @username6: Vaccination rate at Google’s and Pixar’s daycare is less than 50% http://t.co/6GFxs6VDI2 http:/...” (1604 retweets)

Four of these five tweets were about news stories: the first was a reference to a *Forbes* magazine article published on February 4, 2015; the third referred to a CNN story published on February 2; the fourth to a *New York Times* op-ed feature published on February 7; and the fifth to a *Wired* article published on February 11. (Note that some user names and references to some Web links were anonymized in order to protect privacy.) In contrast, the most-retweeted tweet originating from @CDCGov (the Twitter handle of CDC) during that period was “How effective is vaccine against measles? 1 MMR vax dose is ~93% effective at preventing #measles if exposed; 2 doses are ~97% effective.” This was posted on February 9 and was retweeted 182 times during our study period. The average and median retweets per vaccination-related CDC tweet during our study period were 27.9 and 1 respectively.

These statistics suggest that news stories from mainstream media have a substantial impact on health-related social media narratives as well. This is in contrast to official health agencies, which do not appear to have the ability to directly drive these conversations. The importance for the general public of news stories about health issues has been shown before [35], and our data indicate that this holds for social media as well. This finding is in line with other reports [36] that also observed similar patterns in the Netherlands in 2013. Accordingly, an argument is emerging that using such stories to reach the general public offers the potential of higher impact in comparison to direct communications by authoritative official health organizations.

### Communication Patterns: Narrative Structure

**Figure 4:**
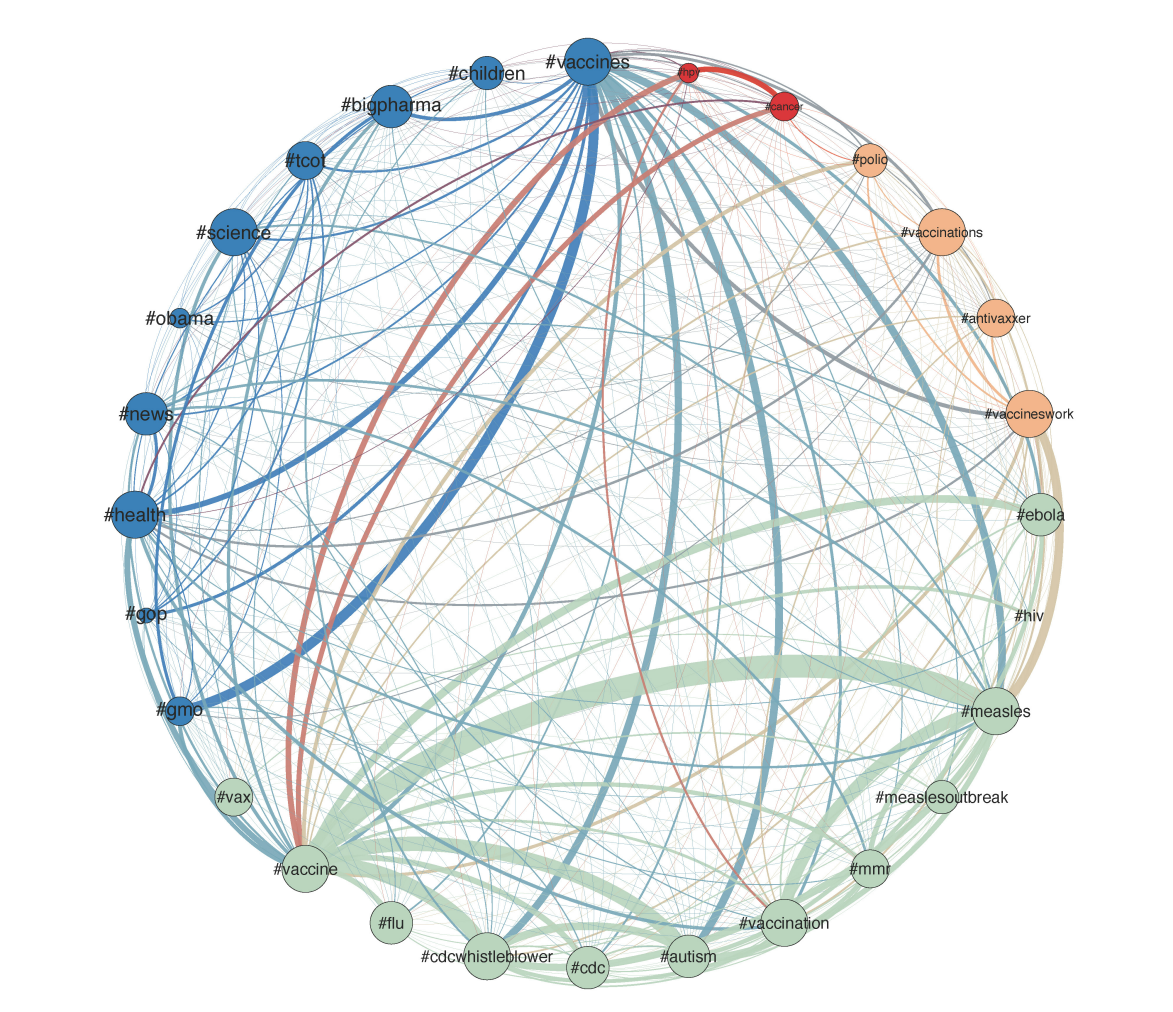
Hashtag associations: clustering based on co-occurrences of hashtags in individual tweets.

The association between words in the data corpus provides additional insights that go beyond mere frequencies. Figure 4 is a visualization of hashtag co-occurrences in tweets. The most frequently encountered hashtags in the data are shown as nodes, with the size of the nodes proportional to their frequencies. The connections between these nodes reflect the frequency of hashtag co-occurrence within single tweets. Every time two hashtags appear together in a single tweet, a connection is established between them. Thicker connecting lines correspond to more frequent co-appearances.

Figure 4 shows how the patterns of co-occurrence of the most popular hashtags can be grouped into four different narrative sets through the application of the Louvain method [37]. We used the Louvain method because it is a data-driven, unsupervised community detection algorithm. As such, this approach does not require an a priori selection of the number of communities (clusters), instead this number emerges through an optimization process. Therefore, it eliminates potential perceptual biases, to maintain a data-driven approach to analyzing these public contributions.

As hashtags have an elevated semantic meaning compared to other words in a tweet, their co-occurrence has been shown to be an important indicator of the sentiment of the crowd [38]. This finding can be extended by arguing that these co-occurrences reflect the contextual association of the corresponding topics/issues by the authors. Accordingly, hashtag co-occurrences reveal the structure of the narrative by showing the distinct themes (as clustered associations of hashtags) that are present in the data corpus. More specifically, the Louvain clustering revealed four communities of words that can be considered distinguishable among our data (see Figure 4). In this figure, the color of a node corresponds to its cluster.

Through this clustering shown in Figure 4, we are able to identify the four key thematic dimensions that characterize the public views of the issue. The blue nodes focus on the political aspects of the vaccination, grouping hashtags such as #vaccines, #gmo, #bigpharma, #news, #obama, #gop, and #tcot (standing for “top conservatives on twitter”). The green nodes connect #vaccine to less overtly political, and more health-oriented issues like #cdcwhistleblower, #mmr, and #autism. The light brown nodes show the narrative cluster reflecting the anti-antivaccination activism, which uses polio (#polio) as an argument in support of vaccination practice (#vaccineswork). The red nodes for HPV and cancer represent a conversation occurring outside the measles epidemic that also touches on vaccine themes.

Links among nodes in Figure 4 indicate how frequently terms co-occur in the data corpus. The strongest link in Figure 4 is for the co-occurrence between #vaccine and #measles, which is expected given that the target dates were selected to capture reactions to a measles outbreak linked to under-vaccination. Taking this co-occurrence as having a strength of 1.00, the second strongest co-occurrence is between #vaccine and #cdcwhistleblower (with a strength of 0.64), followed by #vaccine and #autism (0.62), #vaccination and #measles (0.53), and #vaccines and #gmo (0.43).

The information that is gained from such an analysis is primarily an explicit view of how the public associates different topics in its communications, and as such exposes the meta-meaning of these terms. Some of that information may be expected: it is not surprising that measles and vaccine are indeed highly connected in our data. Nevertheless, it is the ensemble of connections that carries high observational value. For example, observing that the antivaccination views (reflected here through the term #cdcwhistleblower) are clustered within the main health-oriented discussion (green nodes) rather than as a peripheral activist debate topic (brown nodes) shows the success of a grass-roots campaign that has brought this issue to broader view in the context of vaccination. Similarly, the fact that Ebola was clustered within the same green group as measles, and not together with HPV and cancer also signifies the semantic affinity that the general public assigns to two infectious diseases that were recently subjects to outbreaks. The data-driven Louvain approach for clustering is highly suitable for that purpose, as it allows us to derive these associations directly and agnostically, unlike for example a top-down thematic approach (eg, k-means clustering) where such information would have been kept separate under the general term of “other diseases.”

**Figure 5:**
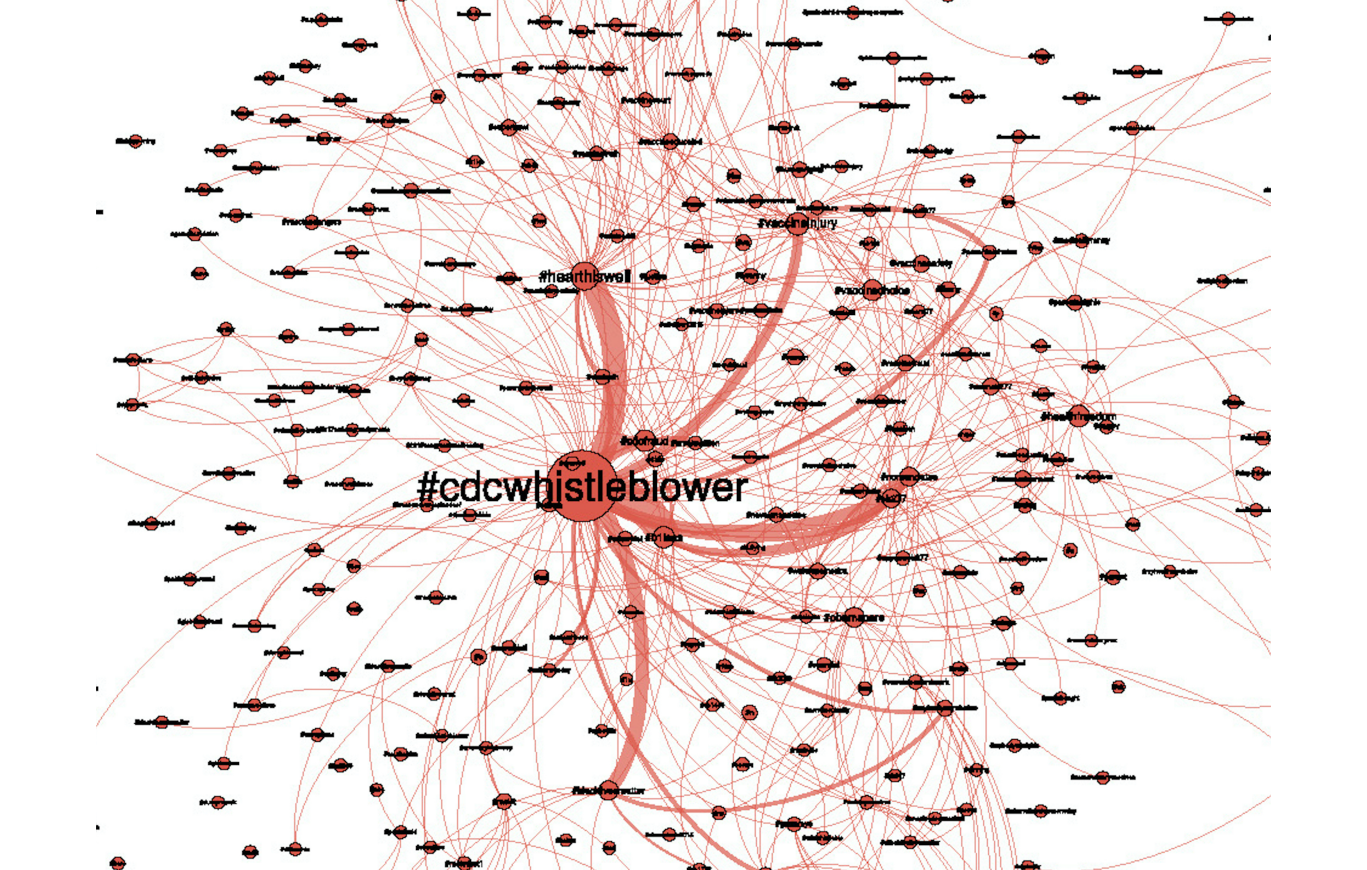
A finer resolution view of the #cdcwhistleblower cluster of Figure 4.

While Figure 4 shows a high-level representation of the themes of the Twitter narrative and some connections among them, the inherently hierarchical structure of this narrative enables further analysis. Figure 5 shows a finer resolution view of the #cdcwhistleblower cluster that was represented as a single node in the hashtag network of Figure 4. Figure 5 uses the same visualization principles as Figure 4: node sizes reflect the frequency of the corresponding hashtag, connections reflect co-occurrence, and the widths of connecting lines represents the frequency of co-occurrences.

The top 10 hashtags associated with #cdcdwhistleblower are shown in Table 3. The first column lists the hashtag itself, while the second column lists the number of times that each hashtag co-occurred with #cdcwhistleblower in the data corpus. The widths of the links among terms in Figure 5 are directly proportional to these numbers. In order to better communicate the level of association among these terms, column 3 of Table 3 lists the percentage of these co-occurrences relative to the overall presence of a particular hashtag. It expresses the ratio of column two over the total number of occurrences of this hashtag in the entire data corpus. This percentage is referred to as the “level of affiliation with #cdcwhistleblower.” For example, #b1less is encountered 2371 times in the same tweets as #cdcwhistleblower, corresponding to 51.48% of all of the tweets in the vaccination data corpus that use the term #b1less. As such, it can be considered as a term with a very high affiliation to the #cdcwhistleblower movement. The same argument can be made for hashtags like #nomandates or #cdcfraud (34.08% and 31.72% respectively). In contrast, #autism, while having a strong presence within the #cdcwhistleblower community (it was encountered 1215 times in conjunction with #cdcwhistleblower) is not exclusive to that discussion, as only 8.97% of its encounters are affiliated with it. These pairwise association strengths communicate the level to which certain arguments are aligned in the context of this health-related argument. Such data analysis processes progressively reveal the complex structure of the health-related narrative in social media, which is essential knowledge in the quest for more effective health communication campaigns.

### From Cyber to Geographical Space

While these social media interactions take place in cyberspace, the communities that participate in them have definitive footprints in the physical space. Accordingly, assessing the geographical patterns of involvement in this discussion provides greater understanding of the motivating factors behind this process. In order to study this, the geolocated tweets from the data corpus were mapped to explore spatial patterns.

**Figure 6:**
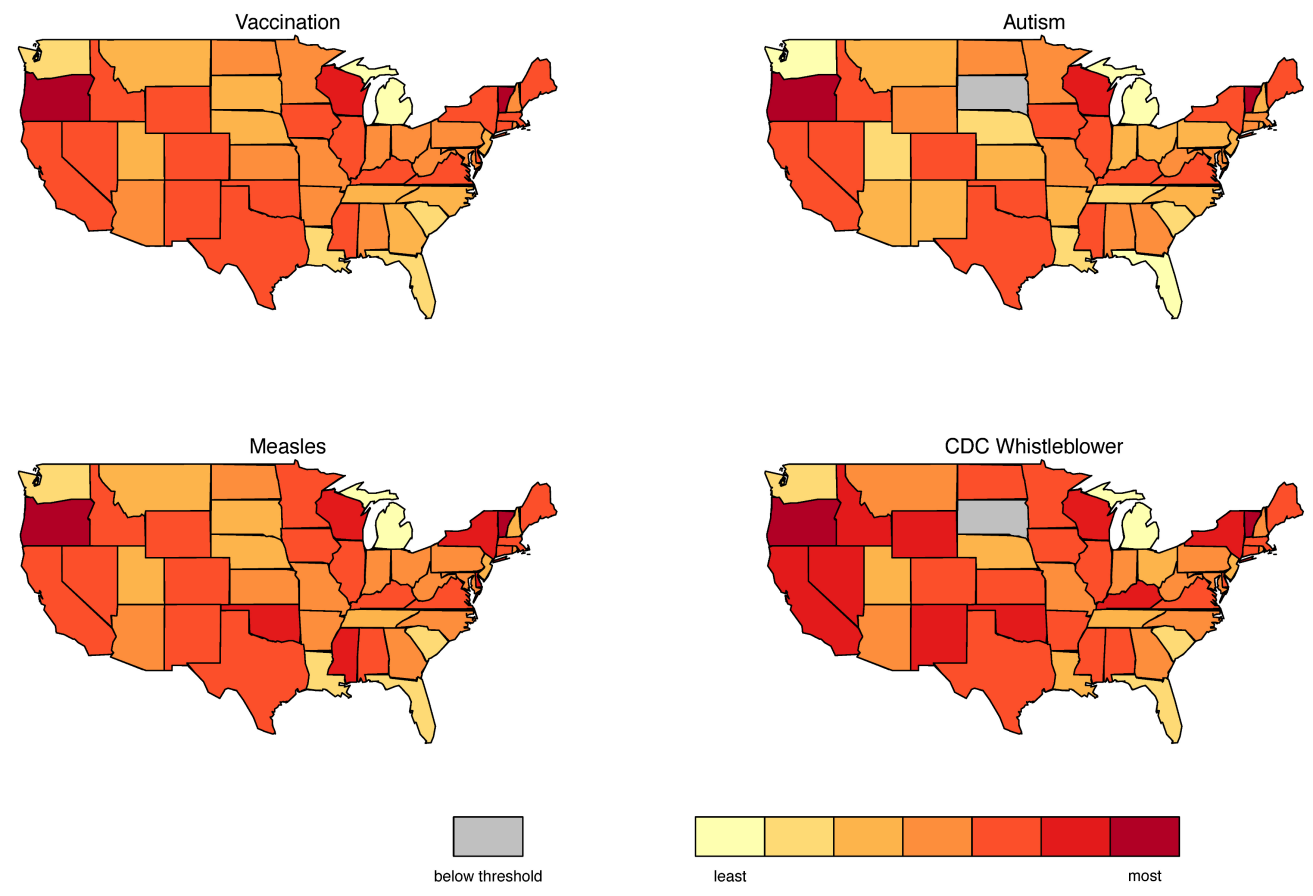
Geographical patterns of participation in the vaccination debate in social media across the contiguous United States.

Figure 6 shows maps of the frequency of tweets mentioning several key terms in the data corpus, aggregated by state. In order to make the data comparable across states, they were normalized by population. The number of tweets originated from within each state were divided by the state population in order to capture the rate of tweets per 10,000 residents for each state. The top left figure communicates the degree of participation in the vaccination debate, expressed as levels of normalized tweets per state. The top right map shows the corresponding metric for references to autism, the bottom left map shows frequency of references to measles, and the bottom right map shows references to #cdcwhistleblower. In these maps, the level of participation is visualized by a color scale, ranging from dark red (highest participation) to light yellow (lowest). The lowest number of tweets per capita (light yellow in top left map) was 1 tweet per 4527 persons in Michigan, the highest rate was 1 tweet per 817 persons in Vermont, and the median was 1 tweet per 1766 residents. Table 4 presents the top five participating states per topic. Participation is expressed in terms of “1 tweet per X persons,” so lower denominators reflect higher levels participation.

**Table 3:** Levels of association of the hashtags most frequently encountered in conjunction with #cdcwhistleblower.

Hashtag	Co-occurrences with #cdcwhistleblower, n	Level of affiliation with #cdcwhistleblower, %
#b1less	2371	51.48
#vaccine	2306	4.41
#hearthiswell	1686	37.65
#autism	1215	8.97
#vaccines	1123	4.01
#measles	1085	4.21
#blacklivesmatter	960	46.13
#nomandates	779	34.08
#vaccineinjury	699	33.10
#cdcfraud	623	31.72
#breakabillion	421	49.36

**Table 4:** Highest levels of participation per state per topic.

Vaccination		Autism		Measles		CDC whistleblower	
State	Participation	State	Participation	State	Participation	State	Participation
VT	1 in 817	OR	1 in 22,410	OR	1 in 6330	VT	1 in 20,194
OR	1 in 849	VT	1 in 24,077	VT	1 in 6660	OR	1 in 20,204
WI	1 in 1100	WI	1 in 33,100	MS	1 in 8268	WI	1 in 24,162
NY	1 in 1270	MS	1 in 34,309	NY	1 in 8892	WY	1 in 26,201
OK	1 in 1329	OK	1 in 36,332	OK	1 in 8955	KY	1 in 27,378

Two states stand out for high levels of involvement: Oregon and Vermont, which are the two states with the highest rates of religious and philosophical exemption from school-entry vaccines nationwide (6.5% and 5.7%, respectively) [39]. Residents of these states are clearly engaging in strong ongoing debates about vaccination that are visible in the partisanship of their social media posts.

We further studied retweeting patterns in order to differentiate between influencers and amplifiers in social media. Influencers are users whose tweets are the most retweeted and as such have a higher impact on the social media community. Vermont and Oregon lead in influence for the terms vaccination and measles (they are the origins of the most retweeted content), which is consistent with the overall traffic data that we have presented in Figure 6 and Table 3. When it comes to autism and #cdcwhistleblower though, while Oregon remains strong, Ohio and Illinois are the two states that follow. Wisconsin, which also features prominently in the levels of participation (Table 4) is emerging as the leading message amplification hotspot, the state that contributes to the dialogue primarily by retweeting other messages. Mississippi and Iowa serve the same role for measles (MS), and autism and #cdcwhistleblower (IA). This allows us to differentiate the role of different communities, separating ones where the message is formed (influencers) from those where the message is amplified.

## Discussion

### Principal Results

This quantitative study of Twitter discourse showed how social media can be used to study public perceptions of health-related issues. The anatomy of the themes and relations that make up this discussion accurately reflected the major public health news items of the day. The data suggest that the social media discourse regarding vaccination reflects a high level of partisanship and ardor (which are typically associated with polarization) among the involved community.

During the observation period in early 2015, references to measles dominated vaccination-related traffic. Preliminary tests of Ebola candidate vaccines and the release of a high-profile research report regarding HPV vaccination were matched by the strong presence of such terms in the data corpus and the corresponding vaccination narrative (Figure 3 and Table 2). Accordingly, our data indicate that the perceived importance for the general public of news stories about health issues also holds for social media as well: news stories drive public participation. This is an important finding for health information communication in the emerging age of social media, which is becoming only more important if we also consider the weak standing of official health organizations in this emerging landscape. The most popular retweets made references to articles published online by major media outlets. However, official public health agencies, such as the CDC, were not as strongly featured in the narrative.

These observations are indicative of the complex notion of authority in the information dissemination landscape of social media. In this particular case, a bottom-up campaign (represented by #cdcwhistleblower) appears to far outweigh the impact of authoritative sources such as the top-down efforts of CDC and WHO. These findings highlight the inherent bottom-up nature of social media communications and the strong potential of such campaigns to support grass-roots activism (eg, [40]). At the same time, the findings highlight the fact that governmental agencies might find that mainstream media coverage of key health issues is more effective at reaching diverse online communities than direct outreach from authorities. This appears to be counter-intuitive at first, with an indirect approach being more effective than a direct one. But it is substantiated once we consider the fact that social capital is a great commodity in social media, and news organizations clearly outweigh the presence of government organizations in that aspect. Until this difference is addressed, our study suggests that it would be advisable to combine such news features with official Twitter posts by government agencies in order to improve health communications.

These same agencies may find social media analysis to be invaluable for providing insights about how popular health narratives are being shaped, as a better understanding of public perception of health issues can lead to more effective communication strategies. The data analysis showed how the narrative can be broken down into subtopics, ranging from politics and policy to specific health issues (Figure 4), exposing the substructure of this narrative. It also captured the associations among terms (Figure 5 and Table 3) to reveal how individual terms form higher level subnarratives. Detailed analysis of the narrative around #cdcwhistleblower showed how certain terms are highly affiliated with it, to form a specific code language for a grass-roots antivaccination dialogue.

A projection of this cyberspace dialogue onto the geographical space (Figure 6) shows that the two states with the highest rates of exemption from mandatory child school-entry vaccines had notably higher rates of engagement in the vaccination discourse on Twitter. This illustrated the spatial nature of online communities, even though they exist in cyberspace. Projecting social media traffic patterns to the corresponding geographical space provides new insights on where particular health issues are hot topics. Such information can therefore be used to devise more targeted awareness campaigns.

While this study has addressed the issue of vaccination in the context of the 2015 measles outbreak, the methodology presented herein is generalizable and could be applied to the study of any health issue that elicits participation in social media. While doing so, we need to remain aware of the fact that public views and opinions are shaped and re-shaped over time, in response to seminal events, or as a result of an ongoing public debate. Accordingly, while the results of our analysis address the discussion at a specific time period, a longitudinal study of the narrative over time would enhance our understanding of the subject and its multiple societal dimensions.

### Limitations

Arguably the two key limitation considerations associated with the analysis of social media relate to the degree to which social media demographics are reflective of the overall community and to the privacy issues behind such analysis.

The demographic profiles of social media users have been evolving, as participation in such platforms has moved well past the point of being a niche practice to become globally adopted. A recent Pew study [41] indicates that while overall approximately three out of four Internet users in the United States are active in social media, there is a certain age bias. More specifically, there is stronger participation in the 18-49 age group (on average 85%) compared to the 50-64 (65%) and 65+ (49%) age groups. Accordingly, in the context of health informatics, when analyzing such data of certain diseases that have a strong demographic profile associated with them, a certain bias may be introduced [19]. Similarly, when studying participation on a global scale, one needs to account for participation variations across different countries and continents. In our study, considering that our regional analysis focused on the United States and that there are no particular demographic profile data associated with the discussion regarding vaccination, an adjustment for age groups would be of little value. If we are to assume that the participants in this discussion are most likely parents of vaccination-age kids and parents of kids who are at risk of infection in a measles outbreak, their majority would most likely fall in the 18-49 age group, corresponding to the highest levels of participation in social media. Subsequent studies of the demographic profiles of individuals who participate in this ongoing debate in the real world would be beneficial for future analyses. Similarly, adjustments for different age groups would also be very appropriate for studies of other health issues, especially ones where the affected communities are highly skewed age-wise.

While social media demographics are expected to become less of an issue in the future, as the adoption of such technologies becomes even more prevalent, the issue of privacy is a topic that will affect such studies. While we are pursuing these newfound opportunities, we have to remain cognizant of the associated privacy issues, in order to ensure the proper use of this public domain information. This challenge exceeds the simple anonymization of such data. A variety of private attributes can easily be revealed through the integrative analysis of multiple datasets, and revealing the identity of social network contributors who may have opted to keep it secret is feasible [42]. The availability of geolocation information further enhances these concerns, as studies have shown that the analysis of human mobility data (eg, cell phone tracks) allows the unique identification of individuals by using as few as four spatiotemporal points in these trajectories, even when coarse geolocation information is made available [43]. Accordingly, the broad range of information that is communicated through social media, an aggregate of location, social connections, and personal views, is accentuating the need for better multi-source anonymization solutions.

### Comparison With Prior Work

Quantitative studies of the patterns and mechanisms of health-related communication in social media have the potential to yield valuable and actionable information about how health knowledge, attitudes, and beliefs are shaped. Our paper is making a contribution toward this goal by presenting a case study and components of a broader emerging analysis framework, pursuing discernible patterns of this narrative across the cyber-physical nexus.

This emerging research direction is still in its early stages, and only recently some studies have examined attitudes about vaccination in social media. Salathé and Khandelwai [44] studied Twitter content to assess the level of polarization between supporters and opponents of swine flu (H1N1) vaccination, in the broader context of digital epidemiology [45]. Their study focused on sentiment analysis and assessed information flow in social networks by studying follower patterns (rather than retweets, which was the case in our study). This showed the high level of polarization in such exchanges, with Twitter users tending to follow other users who share the same sentiments on the topic. Kaptein et al [46] analyzed a data corpus of 12,500 tweets related to the discussion about HPV vaccination in the Netherlands and showed that health-related discussions on Twitter do not drift to other topics. Comparable polarization patterns were observed in a study of Twitter traffic related to a scheduled vote in Chicago on the regulation of electronic cigarettes by Harris et al [47]. This was a small scale study of 683 tweets of a highly localized event.

Odlum and Yoon [48] studied the use of social media during the 2014 Ebola outbreak, using a set of 42,236 tweets to assess the potential benefits of using social media as a real-time outbreak tracking tool. Toward the same goal, Gurman and Ellenberger [49] studied 2616 tweets in the aftermath of the 2010 Haiti earthquake. These preliminary studies further highlight the potential utility of quantitative studies of social media content and health communication.

Our work advances this state-of-the-art by contributing an additional case study that addresses the attitude toward vaccination in the context of a disease outbreak and by pursuing this study as a complex cyber-physical narrative. The term “narrative” is broad in its nature and has been used in the past in the context of health information (eg, a linguistic analysis of YouTube contributions regarding cancer stories [50]). In the context of this study, we position narrative at the intersection of linguistic, social, and geographical networks. Toward that goal, we analyzed text content, spatial patterns of contributions, and retweet patterns. We focused on retweet activities rather than follow patterns, as retweets tend to be more dynamic. As such, retweet patterns can reveal actual impact rather than potential impact (which is the case with follow patterns in social media). For example, @CDCgov has almost half a million followers, but we observed that the actual impact of its tweets is rather limited. Earlier studies [51] had indicated the need for a more strategic approach by health organizations to manage information dissemination. Our work builds on this observation to show the great value of employing news stories to disseminate such information, rather than relying on the direct connection between health organizations and the public. Accordingly, an indirect dissemination avenue (from health organizations to the public through news stories) appears to be more effective than a direct alternative (from health organizations to the public directly).

Furthermore, our paper shows the value of studying this discourse on Twitter as a complex narrative, whereby word associations and the connections between cyber and physical communities reveal the public’s connotations of key issues and actors and the driving forces behind this participation. The fact that we observe strong levels of participation in the social media discourse from states where there is an ongoing debate on vaccination shows the strength of the connections that link the cyber to the physical domains. Examining such connections enables a more comprehensive study of the mechanisms that drive information dissemination and opinion formation in social media. Such findings can be used to design better awareness campaigns and to improve our ability to harvest actionable knowledge from social media data.

### Conclusions

The cyber-physical debate nexus, which connects the cyber narrative in social media to the corresponding geographical space, allows the study of the public’s concerns, views, and responses to health-related issues and thus offers a new avenue for exploring health narratives. As these new mechanisms of discourse are emerging, health communications and health informatics have to adapt to these newfound capabilities and challenges. Advancing our understanding of the mechanisms and patterns of communication in these media is therefore becoming increasingly important. Toward this goal, this study showcased emerging data analysis approaches. These approaches are inherently interdisciplinary, bringing together principles and practices from health informatics, data analytics, and geographical analysis. Further coalescing such capabilities will advance health communication, supporting the design of more effective strategies that take into account public perceptions and concerns. At the same time, we need to remain cognizant of privacy issues associated with the nature of social media communications. Studying the narrative rather than the individuals and aggregating data in geographical spaces can maintain the relevance of the analysis while also preserving user anonymity.

## Abbreviations

API: application program interface
CDC: Centers for Disease Control and Prevention
HPV: human papilloma virus
NIH: National Institutes of Health
WHO: World Health Organization
